# Prognostic impact of the prognostic nutritional index in cases of resected oral squamous cell carcinoma: a retrospective study

**DOI:** 10.1186/s12903-021-01394-6

**Published:** 2021-01-22

**Authors:** Atsushi Abe, Hiroki Hayashi, Takanori Ishihama, Hiroshi Furuta

**Affiliations:** Department of Oral and Maxillofacial Surgery, Nagoya Ekisaikai Hospital, 4-66 Syounen-cho Nakagawa-ku, Nagoya, 454-8502 Japan

**Keywords:** Oral cancer, Systemic inflammation, Biomarkers, Prognosis, Nutritional status

## Abstract

**Background:**

The systemic inflammatory response and nutritional status of patients with malignant tumors are related to postoperative results. We examined the usefulness of the prognostic nutritional index (PNI) as a prognostic tool in patients with oral squamous cell carcinoma who underwent radical surgery.

**Methods:**

From 2008 to 2019, 102 patients (73 males, 29 females; age, 65.6 ± 9.8 years) who visited our hospital and underwent surgical therapy were included in this study. The endpoint was the total survival period, and the evaluation markers included the lymphocyte count and albumin level in peripheral blood obtained 4 weeks preoperatively, age, sex, alcohol consumption, smoking history, site of the tumor, pathological stage, and surgery status. The PNI was calculated using serum albumin levels and the peripheral blood lymphocyte count. The relationship between the PNI and patient characteristics were analyzed using Fisher's exact test. The Kaplan–Meier method was used to evaluate the survival rate. The survival periods were compared using the log-rank method. We evaluated the prognostic factors for overall survival (OS) and disease-free survival (DFS) in a logistic regression model.

**Results:**

The tumor sites included the maxilla (n = 12), buccal mucosa (n = 11), mandible (n = 17), floor of the mouth (n = 9), and tongue (n = 53). The number of patients with stage I, II, III, and IV oral cancers was 28 (27.5%), 34 (27.5%), 26 (33.3%), and 14 (13.7%), respectively. During the observation period, 21 patients died of head and neck cancer. The optimal cut-off PNI value was 42.9, according to the receiver operating characteristic analysis. The proportion of patients with a short OS was lower in those with PNI higher than 42.9, and the 5-year OS in patients with PNI higher and lower than the cut-off value was 62.3% and 86.0%, respectively (P = 0.0105).

**Conclusions:**

The OS of patients with PNI < 42.9 was lower than that of patients with PNI ≥ 42.9. The PNI, which is a preoperative head-to-foot inflammatory marker, can help in estimating the prognosis of oral cancer.

## Background

Although the treatment of oral cancer and the post-treatment quality of life have improved, late metastasis and recurrence are possible complications [1, 2]. The prognostic factors for patients with oral cancer include tumor depth, vascular and neural invasion, cervical lymph node metastasis, and extranodal invasion [3–8]. However, pathological findings and staging alone cannot completely define prognosis. In cancer involving other organ systems, such as gastrointestinal cancer, host-related factors like nutritional indicators and systemic inflammatory responses, are useful in evaluating survival and recurrence, and the prognosis has been reported to relate with these factors [9–19]. The systemic inflammatory response is not only an indicator of the nutritional status [20, 21] but is also useful as a prognostic tool based on mechanisms different from those underlying tumor markers [22]. A previous report has examined the systemic inflammatory response and the effect of the nutritional status in patients with oral cancer receiving radiation or chemotherapy; however, there are few reports on patients having undergone surgical therapy [10, 23].

The prognostic nutritional index (PNI) is evaluated using the serum albumin level and the lymphocyte count. Albumin has been reported as a biomarker of the nutritional status, and its level has been identified to be related to the co-morbidities and the prognosis for certain cancers [24, 25]. It evaluates the susceptibility to infection by assessing malnutrition associated with insufficient protein intake and the evaluation of biological defense capabilities using tests combining evaluation of visceral protein status and immunological function. The lymphocytes take part in cell-mediated immunity and inhibit proliferation and invasion of cancer cells [26]. Therefore, PNI reflects the nutritional status and immunological state of the patient.

The clinicopathologic utility of the PNI has been studied for several malignant tumors, and it has been reported as an independent prognostic tool to assess patient overall survival (OS) [21, 27, 28]. However, the prognostic value of the PNI and its clinicopathologic correlation in patients with oral cancer remains unknown. Therefore, we aimed to examine whether the preoperative PNI could affect the 5-year survival rates in patients who have undergone surgical treatment for oral cancer.

## Methods

### Patients and evaluating parameters

We performed a cross-sectional analysis, including patients with primary oral cancer. We included 102 out of the 117 patients who visited the Nagoya Ekisaikai Hospital and underwent radical surgical therapy for oral squamous cell carcinoma between Jan 2008 and June 2019. Fifteen patients were excluded due to recurrence, metabolic diseases (such as diabetes mellitus), missing data, or the case that treatment was not able to continue because of intention and the overall status of the patients. Data of 102 patients (73 men, 29 women; mean age, 65.6 ± 9.8 years; the Performance Status(PS) intended for the patients of 1 or 2 were analyzed in this study. The clinical and histopathological features and the treatment course of the patients were retrospectively assessed using their medical records. The inclusion criteria for treatment protocol followed were as follows: (1) Extent of resection was determined using a clinical examination, imaging, and evaluation of cervical lymph node metastasis, degree of differentiation, and degree of invasion. (2) Safety margins for resection were kept at 1 cm. (3) Prophylactic neck dissection was not performed for patients without lymph node involvement. However, when the case at elevated risk for the potential metastasis and an ablative range was big, and reconstructive operation was necessary for cT3/T4N0, the dissection of the neck was performed. (4) Neoadjuvant chemotherapy or radiotherapy was not administered. (5) When more than two histopathologically confirmed extracapsular lymph nodes were present or the safety margin of the resection stump was inadequate, postoperative chemoradiotherapy was administered. The average observation period was 48.1 months (6–252.1 months). The examined factors were the survival periods and the long-term prognosis based on PNI grouping. We assessed clinical background factors (preoperative peripheral blood lymphocyte and monocyte counts in relation to age, sex, alcohol consumption history, smoking history, site of the primary tumor, TNM classification, and tumor stage) to examine their association with the OS and DFS (disease-free survival). Lymphocyte and neutrophil counts were measured from peripheral blood samples obtained within 4 weeks before radical surgery. Oral cancer evaluation was based on the findings obtained from visual examination, palpation, computed tomography, and magnetic resonance imaging, and an assessment of the site of occurrence and progression was also performed. Tumor stage was defined according to the Union for International Cancer Control classification [29]. The overall health was evaluated using the body mass index (BMI), albumin levels, and a preoperative examination. The PNI, a systemic inflammation biomarker, was calculated using the serum albumin level and peripheral blood lymphocyte count. The OS was defined as the period between the diagnosis of OSCC and either death. DFS was defined as the time between the first operation to the first documented recurrence, metastasis, or death. Patients, who had not passed away at the end of the investigated period, or patients in whom it was unclear if they had passed away, were censored. The formula used for PNI calculation is as follows [30]:

$$\text{PNI}=\left[ 10\times \text{serum}\text{albumin}\text{level}\left( \text{g/dL} \right) \right]+\left[ 0.005\times \text{total}\text{peripheral}\text{lymphocyte}\text{count}\left( \text{per}\text{m}{{\text{m}}^{3}} \right) \right]$$

The study was approved by the Ethical Review Board of Nagoya Ekisaikai Hospital (approval no. 2019–046), and written informed consent was obtained from all participants.

### Statistical analysis

We conducted a univariate analysis to examine the association of the PNI with the prognosis. Then, we performed multivariate analysis using selected prognosis-related factors. The multivariate analysis was performed by calculating the hazard ratio (HR) and 95% confidence interval (CI) using the Cox proportional hazards model. Patient characteristics and their relationships with the PNI score were analyzed using Fisher's exact test. Analyses of the associations between PNI multiple clinicopathological parameters were conducted using Fisher's exact test or Mann–Whitney U test accordingly.

The PNI cut-off level was set using the receiver operating characteristic (ROC) curve and the area under the curve (AUC) analysis. The ratios of patient OS and DFS were calculated with the Kaplan–Meier method and compared with the log-rank test. Prognostic factors for the OS and DFS were adjusted in a Cox regression model before the evaluations. All analyses were performed with a two-sided test, and P values of 0.05 or less were considered statistically significant. Kaplan–Meier curves of the estimated OS and DFS were generated, and comparisons between the groups were performed using the log-rank test. The multivariate analysis used a Cox proportional hazards model. Each variable was deleted by the model only when the supporting P values in the univariate analysis were 0.1 or higher. All statistical analyses were performed using EZR (Jichi Medical University, Saitama Japan), a graphical user interface for R Ver. 2.8.1 (The R Foundation for Statistical Computing, Vienna, Austria).

## Results

### Clinicopathological characteristics of the patients

**Fig. 1:**
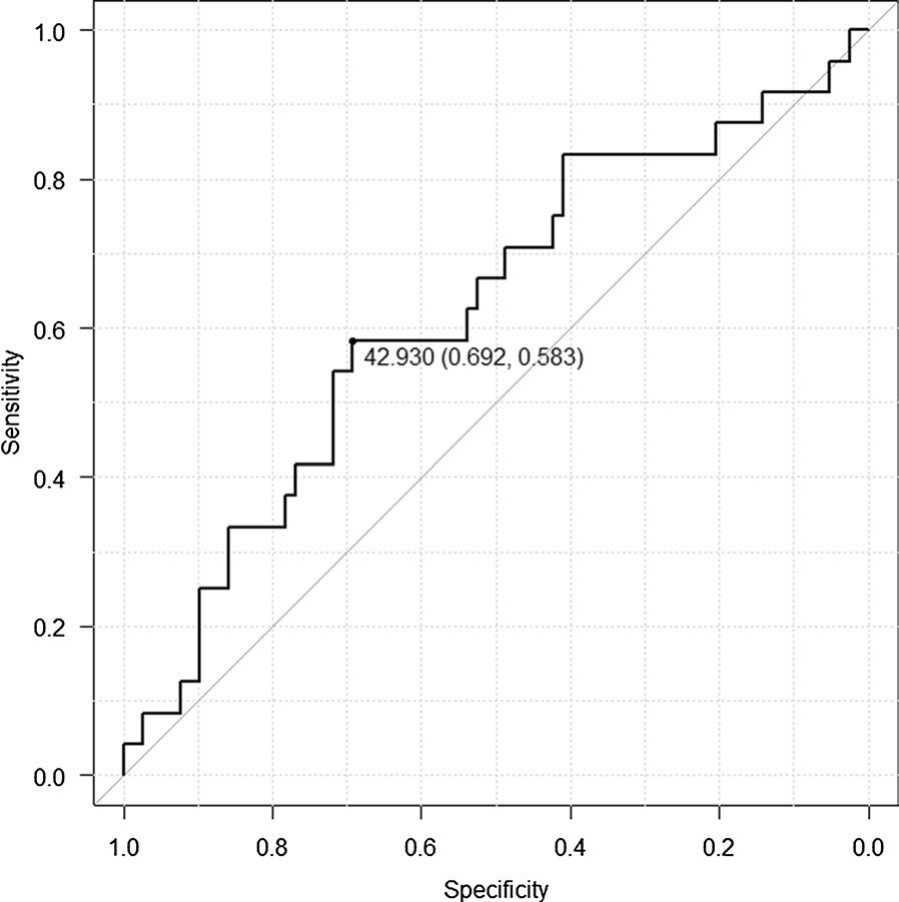
ROC curve for the PNI. The continuous variables PNI and OS were used as the test and state variables, respectively. The PNI cut-off value was 42.9 with the area under the curve, sensitivity, and specificity being 0.634, 0.765, and 0.524, respectively. *ROC* receiver operating characteristic, *PNI* prognostic nutritional index, *OS* overall survival

**Fig. 2:**
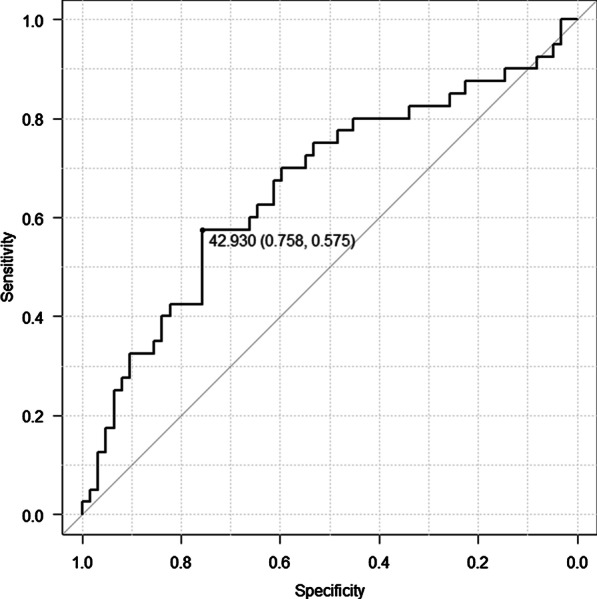
ROC curve for the PNI. The continuous variables PNI and DFS were used as the test and state variables, respectively. The PNI cut-off value was 42.9, with the area under the curve, sensitivity, and specificity being 0.663, 0.758, and 0.575, respectively. *ROC* receiver operating characteristic; *PNI* prognostic nutritional index, *DFS* disease-free survival

Table 1 shows the characteristics of the patients included in this study. The average age of the patients was 65.6 ± 9.8 years, and the number of men and women was 73 (71.6%) and 29 (28.4%), respectively. Sixty patients (58.8%) had a history of smoking. The BMI ranged from 33.5 to 14.9 kg/m^2^ (mean ± standard deviation, 22.8 ± 3.9 kg/m^2^). The PNI ranged from 49.4 to 38.8 (mean, 44.0 ± 2.14). We used a ROC curve analysis to evaluate whether the PNI could predict DFS or OS. ROC analyses showed that the optimal PNI was 42.9 (OS: sensitivity- 69.2, specificity- 0.583; AUC = 0.62; DFS: sensitivity- 75.8, specificity- 0.575; AUC = 0.66) (Figs. 1, 2). The PNI cut-off value was therefore set at 42.9, and the patients were divided into low PNI (< 42.9; OS: n = 37 [36.3%]; DFS: n = 35 [34.3%]) and high PNI (42.9 ≤ ; OS: n = 65 [63.7%]; DFS: n = 67 [65.7%]) groups.

**Table 1:** Characteristics of the patients

Variables (n = 102)	Group	n (%)
Age (mean ± SD)		65.6 ± 9.8
Sex	Male	73 (71.6%)
	Female	29 (28.4%)
Smoking status	Smoker	60 (58.8%)
	Never-smoker	42 (41.2%)
Alcohol	Nondrinker	43 (42.2%)
	Drinker	59 (57.8%)
BMI (mean ± SD)		22.78 ± 3.87
Tumor site	Maxilla	12 (11.8%)
	Buccal	11 (10.8%)
	Mandible	17 (16.7%)
	Floor of mouth	9 (8.8%)
	Tongue	53 (52.0%)
T	1	30 (29.4%)
	2	53 (52.0%)
	3	12 (11.8%)
	4	7 (6.9%)
N	0	75 (73.5%)
	1	22 (21.6%)
	2	5 (4.9%)
Stage	1	28 (27.5%)
	2	34 (33.3%)
	3	27 (26.5%)
	4	13 (12.7%)
Tumor differentiation	Well	52 (51.0%)
	Moderately	41 (40.2%)
	Poorly	9(8.8%)
Lymphovascular invasion	No	70 (68.6%)
	Yes	32 (31.4%)
Vascular invasion	No	97 (95.1%)
	Yes	5 (4.9%)
Perineural invasion	No	92 (90.2%)
	Yes	10 (9.8%)
Close margin (< 5 mm by histopathology)	No	95 (93.1%)
	Yes	7 (6.9%)
Postoperative treatment	No	76 (74.5%)
	Yes	26 (25.5%)
Neutrophil (mean ± SD)		58.89 ± 9.15
Total lymphocytes (mean ± SD)		1857.48 ± 711.19
Alb (mean ± SD)		4.03 ± 0.24
CRP (mean ± SD)		0.35 ± 0.58
PNI (mean ± SD)		44.01 ± 2.14

*BMI* body mass index, *CRP* C-reactive protein, *PNI* prognostic nutritional index

### The OS and DFS, according to the PNI

**Fig. 3:**
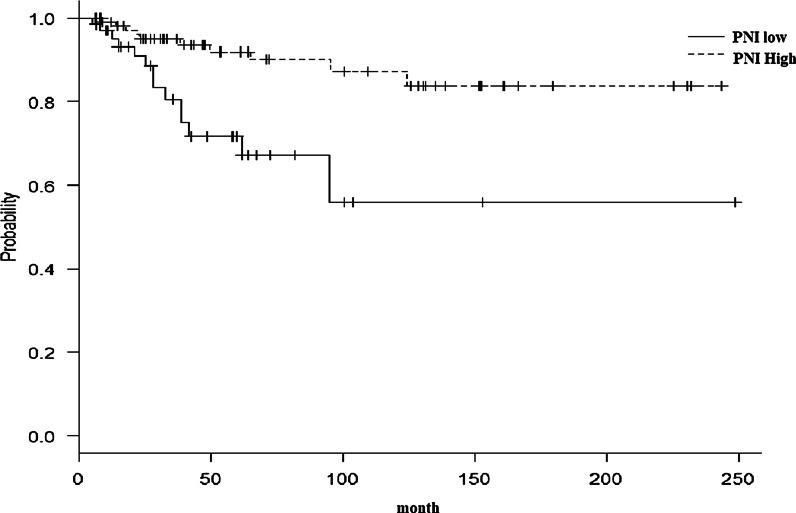
Kaplan–Meier survival curves for the PNI and overall survival of oral squamous cell carcinoma patients. Kaplan–Meier curves, according to the PNI score. The OS was significantly worse in patients with a lower PNI than those with a higher PNI (≥ 42.9) (P = 0.0007886, respectively). *PNI* prognostic nutritional index, *OS* overall survival

**Fig. 4:**
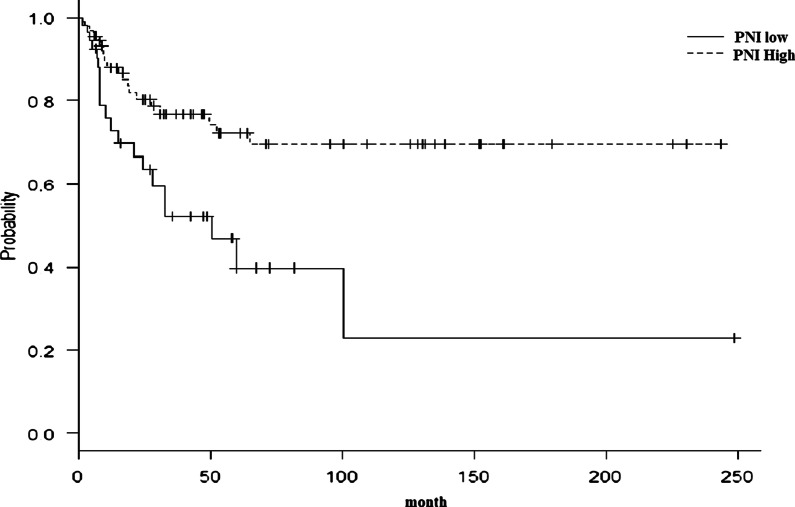
Kaplan–Meier survival curves for the PNI and the DFS of oral squamous cell carcinoma patients. Kaplan–Meier curves, according to the PNI score. The DFS was significantly worse in patients with lower PNI than those with a higher PNI (≥ 42.9) (P = 0.000005792, respectively). *PNI* prognostic nutritional index, *DFS* disease-free survival

The relationship between specific clinicopathological factors and the OS and DFS is summarized in Tables 2, 3. The Kaplan–Meier survival curve outlining the relationship between the PNI and the OS and DFS rate (P < 0.001) is shown in Figs. 3 and 4. The group with low PNI showed significantly lower rates of OS and DFS compared to the group with high PNI. Univariate analysis revealed that the stage (P = 0.016), vascular invasion (P = 0.014), pre-treatment serum CRP level (P = 0.002), and PNI (P = 0.011) were associated with the rate of OS (Table 2); however, univariate analysis revealed no association between the rate of DFS and the stage (P = 0.042), albumin level (P = 0.045), pre-treatment serum CRP level (P = 0.007), lymphovascular invasion (P = 0.001), postoperative treatment (P = 0.0002), and PNI (P = 0.006) (Table 3). As a result of the analysis, multicollinearity was absent. We included the factors included in the univariate analysis along with important prognostic factors (histopathological differentiation, surgical margin, vascular and perineural invasion, and postoperative treatment) as covariates in the multivariate analysis. The multivariate analysis showed that only the CRP level (HR 2.99; 95% CI 11.20–7.46; P = 0.019), perineural invasion (HR 3.73; 95% CI 1.06–13.09; P = 0.04), and PNI (HR 0.32; 95% CI 0.13–0.79; P = 0.013) were associated with the rate of OS (Table 4). The multivariate analysis also showed that the margin (HR 4.10; 95% CI 1.13–14.94; P = 0.032), postoperative treatment (HR 3.71; 95% CI 1.65–8.33; P = 0.0015), and the PNI (HR-0.27; 95% CI 0.13–0.54; P = 0.0024) were independent predictors of the DFS (Table 5).

**Table 2:** Univariate analysis of the associations between the clinicopathological characteristics of the patients and their prognostic variables and overall survival

Variables	Group	n	Survival rate (%)	P value	HR	95% CI
Age	< 66	51	81.4	0.326		
	66 ≧	51	71.7		1.33	0.31–5.64
Sex	Male	73	72.8	0.472		
	Female	29	88.5		3.58	0.41–31.10
Smoking status	Smoker	60	70.5	0.114		
	Never-smoker	42	87.3		0.19	0.03–1.39
Alcohol	Nondrinker	43	89.4	0.101		
	Drinker	59	67.5		1.82	0.33–10.10
BMI	22.4 <	52	75.4	0.977		
	22.4 ≧	50	78.2		1.03	0.29–3.76
T	≦ 2	83	77.8	0.671		
	3 ≧	19	71.8		0.13	0.03–0.64
N	≦ 2	75	76.0	0.646		
	3 ≧	27	77.1		0.03	0.00–0.22
Stage	≦ 2	62	87.1	0.016		
	3 ≧	40	63.5		34.3	5.99–19.640
Grade	Well/moderately	93	76.4	0.399		
	Poorly	9	77.8		0.14	0.02–1.18
Lymphovascular invasion	No	70	81.6	0.22		
	Yes	32	67.9		0.81	0.22–2.94
Vascular invasion	No	97	78.0	0.014		
	Yes	5	60.0		1.73	0.38–7.90
Perineural invasion	No	92	77.8	0.062		
	Yes	10	70.0		6.11	1.02–36.57
Neutrophils	< 59.6	51	80.2	0.092		
	≧ 59.6	51	73.0		4.93	1.21–20.00
Total lymphocytes	< 1730	51	74.7	0.776		
	≧ 1730	51	80.8		1.42	0.44–4.65
Alb	< 4	55	75.0	0.57		
	≧ 4	47	79.1		1.42	0.16–12.92
CRP	< 0.2	59	86.3	0.002		
	≧ 0.2	43	62.8		5.67	1.50–21.49
PNI	< 42.93	35	64.3	0.011		
	≧ 42.93	67	84.1		0.15	0.02–1.14
Postoperative treatment	No	76	80.5	0.069		
	Yes	26	66.5		1.35	0.34–5.39
Close margin (< 5 mm by histopathology)	No	95	76.6	0.433		
	Yes	7	85.7		2.48	0.31–20.02

*BMI* body mass index, *CRP* C-reactive protein, *PNI* prognostic nutritional index

**Table 3:** Univariate analysis of the associations between the clinicopathological characteristics of the patients and their prognostic variables and DFS

Variables	Group	n	Survival rate	P value	HR	95% CI
Age	< 66	51	0.622	0.233		
	66 ≧	51	0.515		1.37	0.55–3.38
Sex	Male	73	0.575	0.678		
	Female	29	0.569		2.16	0.66–7.03
Smoking status	Smoker	60	0.569	0.951		
	Never-smoker	42	0.575		0.6	0.19–1.87
Alcohol	Nondrinker	43	0.599	0.84		
	Drinker	59	0.548		1.12	0.34–3.74
BMI	22.4 <	52	0.54	0.454		
	22.4 ≧	50	0.596		0.44	0.18–1.11
T	≦2	83	0.573	0.674		
	3 ≧	19	0.545		0.23	0.07–0.71
N	≦ 2	75	0.563	0.6		
	3 ≧	27	0.572		0.08	0.02–0.32
Stage	≦ 2	62	0.669	0.0421		
	3 ≧	40	0.449		4.46	1.34–14.81
Grade	Well/moderately	93	0.577	0.0665		
	Poorly	9	0.444		1.15	0.25–5.21
Lymphovascular invasion	No	70	0.644	0.00152		
	Yes	32	0.389		1.04	0.39–2.80
Vascular invasion	No	97	0.585	0.267		
	Yes	5	0.4		0.86	0.22–3.34
Perineural invasion	No	92	0.565	0.554		
	Yes	10	0.6		0.88	0.18–4.33
Neutrophils	< 59.6	51	0.664	0.0897		
	≧ 59.6	51	0.477		1.28	0.56–2.90
Total lymphocytes	< 1730	51	0.508	0.33		
	≧ 1730	51	0.651		0.67	0.28–1.58
Alb	< 4	55	0.478	0.0451		
	≧ 4	47	0.676		0.32	0.10–0.99
CRP	< 0.2	59	0.657	0.00721		
	≧ 0.2	43	0.438		1.53	0.66–3.55
PNI	< 42.93	38	0.378	0.00631		
	≧ 42.93	64	0.67		0.61	0.23–1.61
Postoperative treatment	No	76	0.655	0.000221		
	Yes	26	0.337		3.59	1.29–10.00
Close margin (< 5 mm by histopathology)	No	95	0.582	0.0901		
	Yes	7	0.429		10.97	2.77–43.43

*BMI* body mass index, *CRP* C-reactive protein, *PNI* prognostic nutritional index, *DFS* disease-free survival

**Table 4:** Multivariate analyses for the associations with the OS

Variables	Hazard ratio	95% CI	p value
CRP	3.56	1.41–9.01	0.0073
Grade	0.21	0.04–1.12	0.068
PNI	0.25	0.10–0.66	0.0047
Stage	2.48	0.96–6.40	0.061
Lymphovascular invasion	1.25	0.50–3.11	0.64
Vascular invasion	3.29	0.88–12.20	0.076
Perineural invasion	4.7	1.04–21.25	0.044
Close margin (< 5 mm by histopathology)	2.15	0.37–12.51	0.39
Postoperative treatment	1.45	0.54–3.91	0.46

*CRP* C-reactive protein, *PNI* prognostic nutritional index, *OS* overall survival

**Table 5:** Multivariate analyses for the associations with the DFS

Variables	Hazard ratio	95% CI	p value
CRP	1.75	0.89–3.44	0.11
Grade	1.7	0.52–5.57	0.38
PNI	0.37	0.19–0.73	0.0043
Stage	1.09	0.50–2.38	0.83
Lymphovascular invasion	1.62	0.80–3.29	0.18
Vascular invasion	1.1	0.32–3.79	0.88
Perineural invasion	0.65	0.17–2.52	0.53
Close margin (< 5 mm by histopathology)	4.49	1.30–15.53	0.018
Postoperative treatment	3.08	1.39–6.81	0.0054

*CRP* C-reactive protein, *PNI* prognostic nutritional index, *DFS* disease-free survival

## Discussion

In some studies, PNI has been confirmed as a new prognostic tool for cancer, and a low PNI has been shown to be significantly associated with lower survival for pancreatic cancer, hepatocellular carcinoma, esophageal cancer, gastric cancer, colorectal cancer, renal cell carcinoma, and ovarian cancer [31–37]. In other reports, the cut-off value of PNI used to predict prognosis was 42–47.8. The results of our retrospective analysis showed that low preoperative PNI and high CRP levels were prognostic factors for poorer OS and DFS in patients with oral cancer. In this study, we divided the patients into two groups based on a PNI cut-off value of 42.9 derived from the ROC curve, and we compared the clinical background factors in the two groups. The cut-off value of 42.9 that we used in this study is within the range used in previous studies; therefore, it can be argued that PNI is a practical tool to assess postoperative prognosis [38–40]. In the multivariate analysis, a low PNI, a high CRP level, and perineural invasion were significantly associated with poorer OS. Significant differences were also observed in the HR (Hazard Ratio) with respect to the surgical margin, postoperative treatment, and PNI in the multivariate analysis for DFS. Additionally, the two groups showed differences in the DFS and the 5-year OS. These results suggest that the low PNI group has a poorer preoperative nutritional status and a higher degree of inflammatory response than the high PNI group, resulting in poor prognosis. The PNI, which is estimated using the serum albumin level and the lymphocyte count, reflects the nutritional and immunological state of the patient. Previous studies have reported the PNI as a prognostic factor affecting OS for different malignancies [41–45].

Microenvironmental inflammation affects the growth of tumor cells and promotes angiogenesis and metastasis [46, 47]. The immune system recognizes cancer cells and secretes, as a response, inflammatory cytokines, leading to hypercytokinemia [46–48]. Interleukin-8 (IL-8) and vascular endothelial growth factor (VEGF) are two cancer-associated cytokines. These cytokines cause the resolution of the extracellular matrix and neovascularization. Consequently, growth, invasion, and metastasis of tumors are accelerated. However, it is difficult to easily measure these cytokines [49, 50]. Blood biochemical changes caused by these cytokines can be assessed by measuring inflammatory reaction markers based on the systemic inflammatory reaction. [46–51]. To date, numerous traditional systemic inflammation markers have been reported, including the Glasgow Prognostic Score [52, 53] based on plasma components, the neutrophil-to-lymphocyte ratio [54, 55] derived from the number of blood cells, the lymphocyte-to-monocyte ratio [56, 57], CRP-to-albumin ratio [58], and the PNI [27, 59] based on serum albumin levels and lymphocyte counts. Most of these markers are based on blood cell counts, serum protein level measurement, and the ratios derived from these parameters. Albumin is a significant component of the plasma protein content and reflects the nutritional status, whereas lymphocytes reflect the immunological state; therefore, the ratio of serum albumin level to the lymphocyte count is associated with the survival of patients with cancer [60–62].

Low PNI levels show poor prognosis for oral cancer because the inflammatory cytokines IL-6 and IL-8 increased the number of neutrophils and decreased those of lymphocytes besides enhancing proteolysis [48–51]. Thus, low PNI was considered as an indicator of high inflammatory cytokine levels. The release of cytokines by cancer cells results in a rise in the serum CRP level at the same time. Elevated CRP levels have been reported to be associated with a lower rate of DFS and OS in operable oral cancers [62]. Similarly, some reports have investigated the impact of serum albumin and CRP on the outcome of combination chemoradiotherapy in cases of unresectable head and neck cancers [63]. The association between OS and CRP has been reflected in this study.

The mechanisms underlying the associations between systemic inflammatory response and survival in patients with oral squamous cell carcinoma are not evident. However, using albumin levels and lymphocyte counts, the components used for PNI calculation, cancer cachexia associated with growth factors release, impaired cell-mediated immune response, and angiogenesis can be estimated [64–68]. These mechanisms are complex and include a combination of the factors mentioned above. Therefore, further studies involving metrics such as the PNI, along with an appropriate grading system for it, are necessary to assess its prognostic value in oral cancer. We incorporated the PNI in a prognostic model, and the prospective analysis of this model in a large group of patients was essential to assess the pretreatment risk. In the following paragraphs, we provide some hypotheses to explain why a low PNI level is associated with a poor prognosis for oral cancer.

First, the levels of serum albumin, which is a chief component of plasma proteins, can reflect the nutritional status, while lymphocytes, which can eliminate cancer cells and are important components of the immune system, can reflect the immunological state. Thus, the PNI reflects the nutritional and immunological states of the host and can indicate the prognosis in patients with cancer. Consistent with this, the results of some studies have shown that the PNI, after an adjustment for other risk factors, was an independent prognostic factor for the OS.

Second, a low PNI has been reported to be associated with poorer tumor prognosis (increased depth of tumor, lymph node metastasis, poor TNM staging), and an extensive hematic and lymphatic spread. In the multivariate analysis, a significant association was observed between perineural invasion and OS. Cytokines may promote perineural invasion; however, the relationship between such invasion and the PNI is not clear at the moment. Perineural invasion and its relation to PNI are future research themes in oral squamous cell carcinoma.

Multivariate analysis also showed a significant association of the surgical margin, postoperative treatment, and PNI with the DFS. Therefore, PNI has a role in predicting DFS. Moreover, a low PNI is associated with malnutrition and immunosuppression and may inhibit the success of chemoradiotherapy. In this context, PNI can be thought of as having a prognostic value in predicting DFS.

These results suggest that in evaluating systemic inflammatory response in oral cancer, a blood protein reflects the actual situation rather than the blood cells. This suggestion is consistent with a previously published report [27].

Using clinical background factors including the PNI, we performed single multivariate analyses, including factors that are most related to prognosis, and found that a low PNI value was related to prognosis. These results suggest that the PNI is independent of clinical background and surgical-related factors and that the relationship between the PNI and the prognosis may involve a different mechanism from that associated with tumor markers. These results suggest that PNI can predict the prognosis of oral cancer before surgery.

A limitation of this study is the retrospective analysis of data from a single facility. Additionally, the ROC, when determining the cutoff value was relatively low, affected by a treatment protocol, and the number of samples in this study was likely not sufficient (102 cases). Furthermore, since the median observation period was as short as 48.1 months, an increase in the number of cases and longer observation periods are essential. In cases involving metastasis or inflammation, inflammatory cytokines increase the production of acute-phase proteins such as CRP in the liver and reduce the production of albumin. Therefore, when examining a condition including an inflammatory response and considering the change in nutritional status using biomarkers, it should be assumed that the inflammatory response (CRP and white blood cell count) is normal and does not vary [57]. Whether low PNI is the cause or the effect of tumor progression remains unknown, and additional research is required to elucidate this problem.

The assessments of the PNI are cheaper than those involving tumor markers, and the PNI can be easily calculated using blood samples. Therefore, the PNI can be a prognostic factor for OS and may be a useful long-term marker for evaluating recurrence and metastasis before postoperative chemoradiotherapy and during follow-up. Furthermore, poor nutritional status leads to delay and abandonment of postoperative adjuvant therapy and immunological treatment. Thus, these findings may partially explain the relationship between low OS and low PNI in patients with oral cancer.

## Conclusions

The PNI, a cheaper alternative to tumor markers that can be easily measured using common preoperative blood sampling techniques, can be a prognostic tool to assess the OS. This may partially explain its relationship with the survival period in patients with oral cancer. Moreover, it can be a useful long-term prognostic marker for assessing the recurrence, metastasis, and follow-up assessments. Furthermore, PNI assessments may facilitate the choice between postoperative chemoradiotherapy and adjuvant therapy.

## Data Availability

The raw data are confdential and cannot readily be shared. Researchers need to obtain permission from the Institutional Review Board and apply for access to the data from The Ethics Committee of Nagoya Ekisaikai Hospital.

## Abbreviations

BMI: Body mass index
CRP: C-reactive protein
CI: Confidence interval
HR: Hazard ratio
IL: Interleukin
OS: Overall survival
PNI: Prognostic nutritional index
ROC: Receiver operating characteristic
